# Ferroptosis–pyroptosis crosstalk in neonatal necrotizing enterocolitis: epithelial barrier failure, immune amplification, and precision targeting

**DOI:** 10.3389/fcell.2026.1981457

**Published:** 2026-10-14

**Authors:** Juan He, Yu Gu, Shanshan An, Liwei Yan, Xuhua Song

**Affiliations:** 1 Department of Pediatrics, Affiliated Hospital of Hebei University, Baoding, Hebei, China; 2 Department of Pharmacy, Affiliated Hospital of Hebei University, Baoding, Hebei, China; 3 Department of Gynecology, Affiliated Hospital of Hebei University, Baoding, Hebei, China; 4 Department of Otorhinolaryngology, Affiliated Hospital of Hebei University, Baoding, Hebei, China

**Keywords:** ferroptosis, GPx4, HMGB1, intestinal epithelial barrier, necrotizing enterocolitis, NLRP3 inflammasome, precision targeting, pyroptosis

## Abstract

Necrotizing enterocolitis (NEC) is a rapidly progressive intestinal emergency of preterm infants in which epithelial injury, dysregulated innate immunity, microvascular failure and microbial translocation reinforce one another. Ferroptosis and pyroptosis have emerged as complementary components of this process. Ferroptosis is driven by iron-dependent phospholipid peroxidation and failure of the SLC7A11–glutathione–GPX4 defense system, whereas pyroptosis is executed by inflammasome-activated gasdermins with release of interleukin-1β, interleukin-18 and damage-associated molecular patterns. Recent NEC studies implicate epithelial ferroptosis, inflammasome–GSDMD activation, mitochondrial dysfunction, and immune-cell death programs across epithelial, myeloid, lymphoid, and platelet compartments. Current data support convergence through redox stress, mitochondrial injury, damage-associated molecular patterns (DAMPs), and barrier failure; however, coexistence of ferroptotic and pyroptotic signals should not be interpreted as proven reciprocal crosstalk. Direct causal coupling between these pathways in human NEC remains unconfirmed. This Mini Review integrates epithelial, immune and vascular evidence; evaluates candidate biomarkers including HMGB1, intestinal fatty acid-binding protein, S100A12, DEFA6 and lipopolysaccharide-binding protein; and proposes a precision-targeting framework. Human milk–based supportive strategies are clinically relevant, whereas FXR/ACSL4 modulation, ferroptosis inhibitors, NLRP3/GSDMD blockade, and mitophagy-directed interventions remain predominantly experimental in NEC. Progress will require cell-resolved human validation, longitudinal biomarker panels and developmentally appropriate combination strategies that interrupt the death–inflammation cycle without impairing host defense or intestinal maturation.

## 1 Introduction

Necrotizing enterocolitis (NEC) remains one of the most devastating gastrointestinal disorders of prematurity. It typically develops after enteral feeding and microbial colonization, and can progress within hours from nonspecific feeding intolerance to transmural necrosis, perforation, sepsis and death (Neu and Walker, 2011; Niño et al., 2016). Contemporary cohorts continue to report substantial mortality among infants requiring surgery, together with long-term risks of intestinal failure and neurodevelopmental impairment (Jones and Hall, 2020; Cao et al., 2022). Although prevention through human milk feeding, standardized feeding protocols and infection control has advanced, no pharmacologic treatment specifically interrupts the molecular injury program once NEC has begun (Hackam et al., 2019).

The premature intestine is developmentally primed for excessive innate immune activation. Epithelial Toll-like receptor 4 (TLR4) signaling impairs enterocyte migration and repair, while reciprocal TLR9 signaling can restrain this response (Leaphart et al., 2007; Gribar et al., 2009). Breast milk activates epidermal growth factor receptor-dependent protection, and individual human milk oligosaccharides (HMOs) can improve perfusion or directly inhibit TLR4 signaling (Good et al., 2015; Good et al., 2016; Sodhi et al., 2021). Maternal aryl hydrocarbon receptor activation also transfers protective signals to the newborn intestine (Lu et al., 2021). Importantly, NEC tissue injury is not explained by apoptosis alone; necroptosis has a demonstrable pathogenic role, establishing regulated cell death (RCD) as a central link between barrier failure and inflammation (Werts et al., 2020).

Ferroptosis and pyroptosis are especially relevant because both are inflammatory in context yet biologically distinct. Ferroptosis reflects catastrophic iron-dependent lipid peroxidation, whereas pyroptosis produces gasdermin pores, cytokine release and membrane rupture. This review focuses on their potential crosstalk across epithelial, immune and vascular compartments, the biomarkers that could resolve these processes clinically, and the precision interventions most likely to translate. Unlike prior NEC regulated-cell-death reviews that primarily catalogue individual death programs, this Mini Review focuses on cross-compartment ferroptosis–pyroptosis interfaces, separates demonstrated NEC-specific findings from mechanistically plausible convergence, and links this distinction to biomarker feasibility and precision-targeting priorities. Here, “crosstalk” is reserved for situations in which one pathway or its mediators plausibly influence the other; detection of both pathways in the same disease context is described as coexistence unless directional interaction is experimentally demonstrated.

## 2 Ferroptosis: redox failure across epithelial and immune compartments

Ferroptosis was defined as a non-apoptotic, iron-dependent form of cell death and is governed by the balance between oxidizable phospholipids and cellular antioxidant defenses (Dixon et al., 2012). GPX4 detoxifies phospholipid hydroperoxides using glutathione, whereas ACSL4 enriches membranes with polyunsaturated fatty acids that confer ferroptosis sensitivity (Yang et al., 2014; Doll et al., 2017). The broader network includes system x_t_^−^-mediated cystine uptake through SLC7A11, iron storage and trafficking, lipid remodeling and parallel suppressor pathways (Jiang et al., 2021). Premature infants are plausibly susceptible because antioxidant reserves are limited while oxygen exposure, inflammation, transfusion-related iron, formula lipids and ischemia can increase oxidative pressure.

Recent studies provide direct NEC-specific evidence. Intestinal epithelial farnesoid X receptor (FXR) promotes ACSL4-dependent ferroptosis; lipid peroxides released from ferroptotic epithelial cells suppress ILC3-derived IL-22, linking epithelial death to defective mucosal repair. FXR antagonism, ACSL4 inhibition and ferroptosis inhibition reduced experimental NEC, while circulating FGF19 and lipid peroxides correlated with disease parameters in infants (Zhang Y. et al., 2025). Independent transcriptomic and experimental work identified ACSL4 as a hub connecting ferroptosis with hypoxia, inflammation and immune-cell abundance (Dang et al., 2022).

Ferroptosis also occurs outside the epithelium. Inflammatory macrophages from NEC models display iron overload, ACSL4 induction, GPX4 loss and lipid peroxidation; conditioned media from these cells injures epithelial cells, and ferrostatin-1 attenuates both macrophage inflammation and intestinal damage (Shen et al., 2025). N-acetylcysteine reduces epithelial barrier injury and ferroptotic features through a SESN2-associated mechanism (Gao C. et al., 2025). Ferrostatin-1 has shown protection in neonatal animal models and human NEC-derived organoid systems, strengthening translational plausibility while remaining preclinical (Wang et al., 2026). Human-milk-derived dioleoyl phosphatidylethanolamine restores SLC7A11/GPX4-associated defense and reduces ACSL4-related injury (Chen et al., 2024). L-cystine has been reported to couple ferroptosis suppression to IL-6/STAT3-dependent restoration of Th17/Treg balance (Zhu et al., 2026). In parallel, GPX4-deficient regulatory T cells become ferroptosis-prone in NEC, whereas vitamin E preserves GPX4, Treg viability and immunosuppressive function (Luo et al., 2024).

## 3 Pyroptosis: inflammasome execution and inflammatory propagation

Pyroptosis is a lytic inflammatory death program originally linked to infection and inflammasome activation (Bergsbaken et al., 2009). In the canonical pathway, inflammatory caspases cleave gasdermin D (GSDMD), releasing an N-terminal fragment that forms membrane pores and enables cytokine release and osmotic rupture (Shi et al., 2015; He et al., 2015; Liu et al., 2016). In NEC, exaggerated TLR4 signaling supplies a strong priming signal for NF-κB-dependent expression of NLRP3 and pro-interleukin-1β, while hypoxia, mitochondrial dysfunction, potassium efflux and microbial products can provide activation signals.

Cronobacter sakazakii can intensify experimental NEC through TLR4-dependent NLRP3 activation (Chen et al., 2020). Pharmacologic blockade of NLRP3 reduces acute intestinal injury and may also limit later neuroinflammatory and cognitive consequences in experimental models, supporting the concept that intestinal pyroptotic signaling can propagate beyond the gut (Zhu et al., 2021). Sialylated HMOs suppress the TLR4/NF-κB/NLRP3/caspase-1 axis in NEC tissue and experimental systems (Zhang et al., 2021), whereas sodium butyrate reduces NLRP3-dependent GSDMD activation downstream of TLR4/MyD88/NF-κB (Gao Y. et al., 2025). LCN2 knockdown suppresses IL-17 A-associated pyroptosis in neonatal rats (Chen et al., 2026).

Mitochondrial quality control appears to be a critical checkpoint. Allicin activates PINK1/Parkin-dependent mitophagy, lowers mitochondrial reactive oxygen species and reduces NLRP3, cleaved caspase-1 and N-terminal GSDMD in epithelial, organoid and neonatal rat models (Zhang F. et al., 2025). Pyroptosis may also occur in circulating platelets: GSDMD-dependent platelet pyroptosis promotes exosome release and neutrophil extracellular trap formation through mitochondrial ROS and oxidized mitochondrial DNA, and disulfiram-mediated GSDMD inhibition improves experimental NEC (Zhang et al., 2026). Pulmonary epithelial TLR4 activation provides an additional mechanism through which intestinal inflammation can contribute to remote lung injury (Jia et al., 2016).

## 4 A ferroptosis–pyroptosis crosstalk framework

Direct reciprocal molecular crosstalk between ferroptosis and pyroptosis has not been demonstrated in human NEC. The evidence instead supports several plausible interfaces through shared stress signals and tissue injury (Figure 1). First, ferroptotic lipid peroxides and membrane damage can generate DAMPs, impair epithelial repair and expose the lamina propria to microbial TLR ligands. Second, mitochondrial ROS and oxidized mitochondrial DNA can simultaneously worsen lipid peroxidation and activate inflammasome signaling. Third, pyroptotic release of interleukin-1β, interleukin-18 and HMGB1 recruits and activates myeloid cells, increasing oxidative and iron-handling stress. Fourth, macrophage ferroptosis and platelet pyroptosis create paracrine signals that injure epithelial and vascular compartments rather than remaining cell-autonomous events.

**FIGURE 1:**
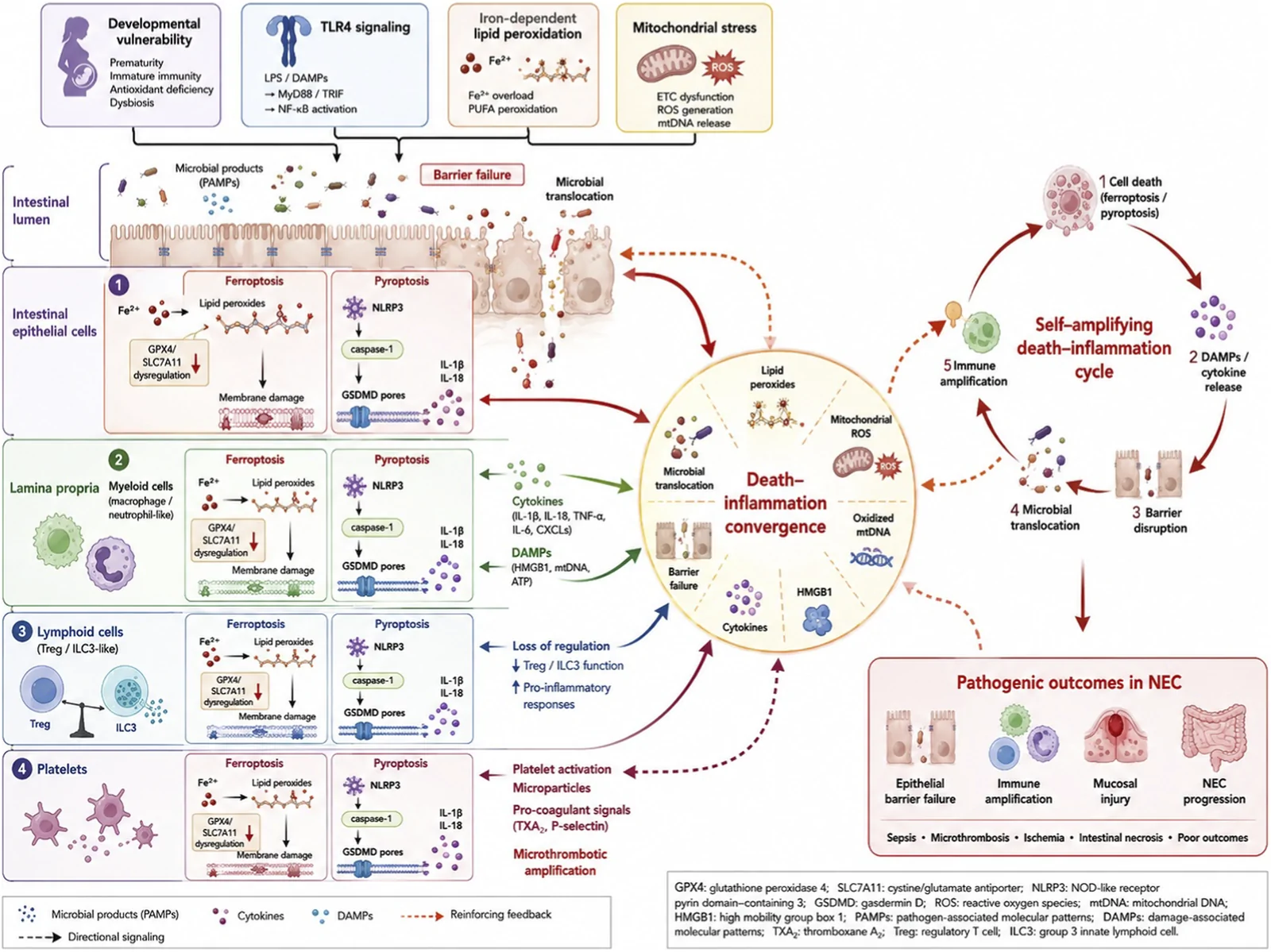
Proposed ferroptosis–pyroptosis crosstalk in neonatal necrotizing enterocolitis. Developmental vulnerability, TLR4 signaling, iron-dependent lipid peroxidation and mitochondrial stress can activate ferroptotic and pyroptotic modules in epithelial, myeloid, lymphoid and platelet compartments. Their convergence through lipid peroxides, mitochondrial reactive oxygen species, oxidized mitochondrial DNA, HMGB1, cytokines, barrier failure and microbial translocation may generate a self-amplifying death–inflammation cycle. Abbreviations: TLR4, Toll-like receptor 4; LPS, lipopolysaccharide; PAMPs, pathogen-associated molecular patterns; DAMPs, damage-associated molecular patterns; MyD88, myeloid differentiation primary response 88; TRIF, TIR-domain-containing adapter-inducing interferon-β; NF-κB, nuclear factor kappa B; Fe^2+^, ferrous iron; PUFA, polyunsaturated fatty acid; ETC., electron transport chain; ROS, reactive oxygen species; mtDNA, mitochondrial DNA; GPX4, glutathione peroxidase 4; SLC7A11, cystine/glutamate antiporter light chain; NLRP3, NOD-like receptor family pyrin domain-containing 3; GSDMD, gasdermin D; IL, interleukin; TNF-α, tumor necrosis factor alpha; CXCL, C-X-C motif chemokine ligand; HMGB1, high-mobility group box 1; ATP, adenosine triphosphate; TXA_2_, thromboxane A_2_; Treg, regulatory T cell; ILC3, group 3 innate lymphoid cell.

An epithelial cell undergoing ferroptosis can reduce ILC3–IL-22-mediated regeneration, while neighboring macrophages or platelets amplify injury through inflammasomes, exosomes and neutrophil extracellular traps. Barrier loss can then increase microbial translocation and TLR4 stimulation, creating a self-reinforcing injury–inflammation cycle. The framework also predicts spatial heterogeneity: ferroptotic epithelium, ferroptosis-resistant surviving cells, pyroptotic immune cells and relatively preserved repair niches may coexist within the same bowel segment. Accordingly, bulk tissue measurements can obscure the cell of origin and the temporal order of events.

HMGB1 is a particularly attractive bridge because it is both a DAMP released during membrane-disruptive injury and a clinically measurable fecal marker. Fecal HMGB1 rises in early NEC, although current evidence is strongest for suspected or early-stage disease and requires validation across severe surgical phenotypes (Vitali et al., 2021). Mechanistically, HMGB1 can be released during membrane-disruptive injury and amplify TLR4/RAGE–NF-κB signaling, thereby linking tissue damage to inflammasome priming; its detectability in stool also makes it more accessible than many research-grade intracellular ferroptosis or pyroptosis markers.

## 5 Biomarkers for mechanism-informed stratification

No single biomarker can currently distinguish ferroptosis from pyroptosis at the bedside. Intestinal fatty acid-binding protein (I-FABP) reflects enterocyte injury and rises in plasma and urine before or during NEC; higher values can identify complicated disease, but kinetics, renal handling and intestinal maturity affect interpretation (Schurink et al., 2015; Gregory et al., 2014). Fecal S100A12 reports phagocyte activation and can rise before severe NEC, although interindividual variability limits stand-alone screening (Dä et al., 2012). Reduced Paneth-cell DEFA6, with preserved GUCA2A, suggests impaired antimicrobial defense in resected NEC tissue (Hoffsten et al., 2023). Lipopolysaccharide-binding protein has recently shown diagnostic and severity-associated performance, especially when combined with C-reactive protein, but prospective neonatal validation is still needed (Zou et al., 2026).

A mechanism-informed panel should combine compartments rather than accumulate nonspecific inflammatory tests, as summarized in Table 1. A plausible design would include: an epithelial-injury marker such as I-FABP; a DAMP such as fecal HMGB1; lipid-peroxidation or ferroptosis-defense measures such as 4-hydroxynonenal, oxidized phospholipids, GPX4 or ACSL4; inflammasome/pyroptosis readouts such as cleaved GSDMD, caspase-1, interleukin-18 or ASC specks; and clinical context including gestational age, transfusion exposure, feeding, perfusion and microbiology. Serial sampling is essential because ferroptotic stress may precede overt inflammatory rupture, whereas pyroptotic and NET-associated signals may rise during progression. Biomarkers should be evaluated for added value over Bell staging, imaging and routine laboratory variables, not merely for case-control significance.

**TABLE 1:** Mechanistic modules, candidate biomarkers and precision-targeting opportunities in neonatal NEC.

Module	Predominant compartment	Representative NEC evidence	Candidate readouts	Precision opportunity/status
Developmental TLR4 priming	Immature epithelium and microvasculature	TLR4 impairs repair; HMOs and breast-milk EGFR signaling are protective (Leaphart et al., 2007; Gribar et al., 2009; Good et al., 2015; Good et al., 2016; Sodhi et al., 2021)	TLR4/NF-κB activity; perfusion; feeding exposure	Human milk/HMOs: clinically plausible prevention; pathway inhibitors remain preclinical
Epithelial ferroptosis	Intestinal epithelial cells	FXR–ACSL4 signaling, SLC7A11/GPX4 failure and lipid-peroxide release impair barrier repair and ILC3–IL-22 signaling (Zhang Y. et al., 2025; Dang et al., 2022; Gao C. et al., 2025; Wang et al., 2026; Chen et al., 2024; Zhu et al., 2026)	ACSL4, GPX4, SLC7A11, 4-HNE, oxidized phospholipids, FGF19	FXR/ACSL4 modulation, redox support and radical-trapping antioxidants; preclinical
Immune-cell ferroptosis	Inflammatory macrophages and Treg cells	Macrophage ferroptosis injures epithelium; GPX4-low Treg ferroptosis weakens immune restraint (Shen et al., 2025; Luo et al., 2024)	Macrophage lipid peroxidation; Treg GPX4; ferritin/iron context	Cell-state-selective ferroptosis inhibition; vitamin E and Fer-1-like strategies require neonatal validation
NLRP3–GSDMD pyroptosis	Epithelium, lamina-propria immune cells	TLR4/NLRP3/caspase-1/GSDMD activation is reduced by SHMOs, butyrate and mitophagy restoration (Chen et al., 2020; Zhu et al., 2021; Zhang et al., 2021; Gao Y. et al., 2025; Chen et al., 2026; Zhang F. et al., 2025)	NLRP3, ASC/PYCARD, cleaved caspase-1, GSDMD-N, IL-18, IL-1β	NLRP3/GSDMD blockade or PINK1/Parkin restoration; infection and developmental safety unresolved
Platelet–NET amplification	Platelets and neutrophils	GSDMD-dependent platelet pyroptosis releases exosomes that promote NETs through mtROS/ox-mtDNA (Zhang et al., 2026)	Platelet caspase-1/GSDMD, exosomes, MPO-DNA, citrullinated histone H3	GSDMD/NET modulation; early translational stage
DAMP and barrier propagation	Stool, plasma and damaged bowel	HMGB1 and enterocyte injury markers rise in NEC; microbial translocation reinforces TLR4 signaling (Vitali et al., 2021; Schurink et al., 2015; Gregory et al., 2014; Dä et al., 2012; Hoffsten et al., 2023; Zou et al., 2026)	Fecal HMGB1, plasma/urine I-FABP, S100A12, LBP, DEFA6	Longitudinal multimarker panels for diagnosis and treatment timing; clinical validation needed
Repair and microvascular rescue	Macrophage–endothelial–epithelial niche	Macrophage-derived IGF-1 and recombinant IGF-1/IGFBP3 preserve microvasculature and repair (Yan et al., 2022; Yan et al., 2024)	IGF-1, endothelial proliferation, perfusion indices	Trophic repair strategies may complement death-pathway modulation; preclinical

## 6 Precision targeting: prevention, rescue and combination strategies

Near-term clinical translation should prioritize established supportive measures that reinforce endogenous intestinal protection. Human milk feeding remains the best-supported nutritional strategy; HMOs can promote epithelial regeneration and inhibit TLR4-related inflammatory signaling (Zito et al., 2024). Human milk also provides antioxidant, immunologic, and lipid mediators, while oxidative stress is increased in infants with NEC (Aydemir et al., 2011; Aceti et al., 2018). Bioactive milk lipids such as DOPE are mechanistically promising, but dose, formulation, and developmental safety remain experimental questions.

Ferroptosis-directed approaches include FXR or ACSL4 modulation, restoration of SLC7A11–glutathione–GPX4 activity, iron and lipid-peroxide control, and radical-trapping antioxidants. Ferrostatin-1 remains an experimental NEC intervention supported by preclinical models and is not an approved neonatal therapy. N-acetylcysteine, vitamin E and cystine-related metabolic support have greater clinical familiarity, yet the doses and redox consequences required to alter NEC-specific ferroptosis cannot be inferred from nutritional use. Moreover, indiscriminate antioxidant therapy could interfere with physiological redox signaling and host defense.

Any precision-targeting strategy must preserve the microvasculature and repair niche. Macrophage-derived IGF-1 promotes neonatal intestinal endothelial proliferation and reduces NEC susceptibility, while recombinant IGF-1/IGFBP3 preserves microvascular integrity, enhances epithelial repair and reduces experimental injury (Yan et al., 2022; Yan et al., 2024). These observations argue for cell-state-specific modulation rather than lineage-wide macrophage depletion: inflammatory macrophage ferroptosis may be pathogenic, whereas trophic macrophage states support vascular growth and epithelial repair. For NEC, the near-term framework should prioritize human-milk-based prevention, biomarker-guided escalation, cell-state-aware targeting, and combinations that preserve antimicrobial competence (Scheese et al., 2023).

## 7 Discussion and research priorities

The emerging evidence reviewed here supports ferroptosis and pyroptosis as two biologically distinct but potentially convergent components of NEC pathogenesis. NEC-specific studies have identified epithelial FXR–ACSL4 signaling, loss of SLC7A11–GPX4-mediated antioxidant defense, ferroptotic inflammatory macrophages, and GPX4-deficient regulatory T cells as relevant components of the ferroptotic response (Zhang Y. et al., 2025; Dang et al., 2022; Gao C. et al., 2025; Wang et al., 2026; Chen et al., 2024; Zhu et al., 2026; Shen et al., 2025; Luo et al., 2024). In parallel, TLR4–NLRP3–caspase-1–GSDMD activation, impaired PINK1/Parkin-dependent mitophagy, and platelet pyroptosis coupled to neutrophil extracellular trap formation provide complementary evidence for pyroptotic and inflammasome-mediated injury (Chen et al., 2020; Zhu et al., 2021; Zhang et al., 2021; Gao Y. et al., 2025; Chen et al., 2026; Zhang F. et al., 2025; Zhang et al., 2026). These findings support a model in which oxidative membrane injury and inflammasome activation coexist within the same disease process. However, they do not yet demonstrate that ferroptosis directly triggers pyroptosis, or vice versa, in human NEC.

The most plausible interface between the two pathways is therefore a shared injury environment rather than a simple linear signaling cascade. Ferroptotic lipid peroxidation can aggravate epithelial barrier disruption and increase exposure of the lamina propria to microbial products, while mitochondrial reactive oxygen species and oxidized mitochondrial DNA can promote both redox injury and inflammasome activation (Zhang Y. et al., 2025; Shen et al., 2025; Luo et al., 2024; Zhang F. et al., 2025; Zhang et al., 2026). Conversely, pyroptosis-associated release of IL-1β, IL-18 and damage-associated molecular patterns can amplify myeloid recruitment, mitochondrial stress and tissue oxidation. HMGB1 is particularly relevant in this context because it links membrane-disruptive injury with innate immune activation and is measurable in stool during early NEC (Vitali et al., 2021). This convergence model is consistent with the broader biology of NEC, in which developmental TLR4 hypersensitivity, impaired perfusion, microbial dysbiosis and defective repair act together rather than through a single dominant pathway (Leaphart et al., 2007; Gribar et al., 2009; Good et al., 2015; Good et al., 2016; Sodhi et al., 2021; Werts et al., 2020; Scheese et al., 2023).

Translation to clinical practice remains limited by the gap between experimental mechanisms and bedside measurements. Most ferroptosis- and pyroptosis-specific markers, including ACSL4, GPX4, SLC7A11, cleaved GSDMD and ASC-related readouts, currently depend on tissue, specialized molecular assays or experimental platforms. In contrast, I-FABP, fecal HMGB1, S100A12, DEFA6 and lipopolysaccharide-binding protein have more direct human evidence as markers of epithelial injury, innate immune activation or disease severity (Vitali et al., 2021; Schurink et al., 2015; Gregory et al., 2014; Däbritz et al., 2012; Hoffsten et al., 2023; Zou et al., 2026). Their major limitation is specificity: none can independently identify ferroptosis or pyroptosis. Future biomarker studies should therefore test longitudinal multimarker panels rather than isolated analytes and determine whether mechanistic markers provide incremental information beyond gestational age, feeding history, Bell stage, imaging and routine laboratory indices. Multicenter prospective neonatal cohorts will be essential because biomarker kinetics may differ according to prematurity, renal function, transfusion exposure, medical versus surgical NEC and major neonatal comorbidities.

Therapeutic translation should likewise distinguish established supportive strategies from experimental pathway inhibition. Human milk and human milk oligosaccharides already have a strong biological and clinical rationale for NEC prevention and can modulate TLR4 signaling, epithelial repair and inflammatory responses (Good et al., 2015; Good et al., 2016; Sodhi et al., 2021; Zhang et al., 2021; Aydemir et al., 2011; Aceti et al., 2018; Zito et al., 2024). In contrast, ferrostatin-1, FXR/ACSL4 inhibition, MCC950, TAK-242, disulfiram and pharmacologic manipulation of mitophagy remain predominantly preclinical (Zhang Y. et al., 2025; Wang et al., 2026; Chen et al., 2024; Zhu et al., 2026; Shen et al., 2025; Luo et al., 2024; Chen et al., 2020; Zhu et al., 2021; Zhang et al., 2021; Gao Y. et al., 2025; Chen et al., 2026; Zhang F. et al., 2025; Zhang et al., 2026). Their neonatal application requires particular caution because excessive suppression of inflammasome or myeloid signaling may compromise antimicrobial defense in extremely premature infants. Similarly, macrophages should not be treated as a uniformly pathogenic lineage: macrophage-derived IGF-1 supports intestinal microvascular development and tissue repair in experimental NEC (Yan et al., 2022; Yan et al., 2024). These observations favor cell-state-specific and stage-specific intervention rather than broad inhibition of entire immune-cell populations.

Future research should define the cell-type-specific distribution of ferroptotic and pyroptotic stress across different stages of NEC, particularly within epithelial, myeloid, lymphoid, and platelet compartments. Longitudinal, low-volume biomarker panels are needed to determine whether the transition from reversible oxidative stress to self-amplifying inflammatory injury can be identified before irreversible intestinal damage occurs. Spatial transcriptomics, single-cell profiling, lipidomics, neonatal organoid models, and multicenter prospective cohorts will be important for resolving these questions and for translating regulated-cell-death biology into clinically useful stratification and treatment strategies.

## 8 Conclusion

Ferroptosis and pyroptosis represent distinct but potentially convergent forms of regulated cell death that contribute to epithelial barrier disruption, immune amplification, and tissue injury in neonatal NEC. Current evidence supports the involvement of epithelial FXR–ACSL4 signaling, SLC7A11–GPX4 redox failure, macrophage and Treg ferroptosis, TLR4–NLRP3–GSDMD activation, defective mitophagy, and platelet–NET signaling, although direct pathway-to-pathway crosstalk in human NEC remains insufficiently demonstrated. The most plausible model is therefore a multicellular death–inflammation network in which oxidative stress, mitochondrial dysfunction, DAMP release, microbial translocation, and inflammasome activation reinforce one another across epithelial, immune, and vascular compartments.
